# Semen Microbiome, Male Infertility, and Reproductive Health

**DOI:** 10.3390/ijms26041446

**Published:** 2025-02-09

**Authors:** Dimitra Chatzokou, Ermioni Tsarna, Efstathia Davouti, Charalampos S Siristatidis, Smaragdi Christopoulou, Nikolaos Spanakis, Athanasios Tsakris, Panagiotis Christopoulos

**Affiliations:** 1 2nd Department of Obstetrics and Gynecology, Faculty of Medicine, “Aretaieion” Hospital, National and Kapodistrian University of Athens, 11528 Athens, Greece; 2 AlfaLab, Hellenic HealthCare Group, 11524 Athens, Greece; 3 Microbiology Department, Faculty of Medicine, National and Kapodistrian University of Athens, 11527 Athens, Greece

**Keywords:** microbiome, sperm microbiome, semen microbiome, male infertility, fertility, metagenomics, next-generation sequencing, NGS

## Abstract

The semen microbiome, once believed to be sterile, is now recognized as a dynamic ecosystem containing a diverse range of microorganisms with potential implications for male fertility and reproductive health. We aimed to examine the relationship between the semen microbiome, male infertility, and reproductive outcomes, highlighting the transformative role of next generation sequencing techniques and bioinformatics in exploring this intricate interaction, and we present a critical review of the published literature on this issue. Current evidence suggests a complex association between the composition of the semen microbiome and male fertility, with certain bacterial genera, such as *Lactobacillus* and *Prevotella* that exert opposing effects on sperm quality and DNA integrity. In addition, the influence of the semen microbiome extends beyond natural fertility, affecting assisted reproductive technologies and pregnancy outcomes. Despite considerable progress, challenges remain in standardizing methodologies and interpreting findings. In conclusion, we identify the lack of a definitive management proposal for couples presenting with this phenomenon, and we underline the need for an algorithm and indicate the questions raised that point toward our goal for a strategy. Continued research is essential to clarify the role of the semen microbiome in male reproductive health and to advance the development of personalized fertility management approaches.

## 1. Introduction

Research into the human microbiome, which encompasses the vast array of microorganisms (bacteria, viruses, fungi, archae, and protozoa) normally colonizing the human body (for the purpose of this review, we will focus on the reproductive system), has become a major focus in recent years. Advances in next-generation sequencing techniques and bioinformatics are now expanding our knowledge about the composition of these microbial communities, while researchers are trying to reveal their roles in health and disease [1,2,3,4]. Several studies and reviews are now discussing the role of microbiota in endometrium and semen [5,6,7,8]. In addition, emerging fields like metagenomics and proteomics offer new tools for analyzing microbial communities and identifying biomarkers related to health outcomes, including aspects and outcomes related to male reproductive health [9,10].

Recent publications have highlighted the importance of the semen microbiome in relation to the male reproductive system and fertility [7,8,11,12]. The presence of microbes in semen does not necessarily indicate infection or disease, since the balanced microbiome is a natural component of the human body. Sequencing methods have consistently shown that human semen is not sterile but harbors specific microbial species, whose functions and origins are not always clear [12,13,14]. Furthermore, some more recent studies are discussing the role of urinary microbiomes in the composition of the semen microbiota [15,16], although there is always a risk of contamination, depending on the sample collection technique [15]. Although some studies have not found significant differences between infertile and healthy men regarding bacterial genera, bacteriospermia leading to dysbiosis of the semen microbiome is more prevalent among infertile individuals [16]. Apart from the effects of dysbiosis, bacteria that induce specific pathological changes in semen, including those resulting from cytokines, reactive oxygen species (ROS), toxins, and bacterial enzymes, can adversely affect sperm and spermatogenesis [17]. In addition, imbalances in the semen microbiome can lead to local inflammation, anatomical changes in the genital tract, and alterations in sperm structure and function [18]. Moreover, the semen microbiome is believed to play a role in immune responses and sperm antigenicity [19], potentially impacting fertility and pregnancy rates [20]. Lastly, the exchange of microorganisms between sexual partners [21] further underscores the relevance of the semen microbiome for reproductive health and offspring well-being [22].

This study aims to present a thorough review of the respective literature, while attempting a critical evaluation of the options that should be presented. This review evaluates the recent literature on the role of semen microbiome in male infertility and reproductive health in humans. In this context, we underline the questions raised above and outline the possible scenarios. Taking into consideration the revolutionary impact of molecular diagnostic techniques on our understanding of the semen microbiome and its function, data arising from studies using only culture-dependent methods will not be analyzed in this review (Supplementary Materials).

## 2. The Composition and Role of the Semen Microbiome

As of now, several research groups have examined the composition and role of the semen microbiome. The conducted studies have examined the semen microbiome in fertile men, its relation to infertility, microbiome aberrations linked to sperm DNA fragmentation, interaction with the female reproductive system, and finally the associations of semen microbiome characteristics with outcomes of assisted reproduction techniques.

### 2.1. Semen Microbiome in Fertile Men

Traditionally, microbiological methods such as cultivation under aerobic conditions were used to study the semen microbiome. Based on their results, it was assumed that the semen from healthy men was either completely devoid of bacteria or contained them only sporadically [14]. However, advances in molecular diagnostics and next-generation sequencing (NGS) have revealed that semen harbors a diverse range of bacteria, including bacteria that are non-cultivable and bacteria that are present in very low concentrations [21,23,24].

Recent findings suggest that the semen microbiome consists of a specific set of microorganisms, but their exact origin remains unclear [25]. It has been proposed that the semen microbiome may originate from infections in the urinary tract, blood, intestines, or vagina [26]. However, the presence of bacterial DNA is not always equivalent to infection and cannot distinguish between transient and chronic infections [21]. A study by Lundy et al. [23] comparing bacterial colonies identified through 16S rRNA sequencing in semen, urine, and rectal swabs of healthy men found that only 2.3% of the standardized species were shared across all three environments, while 10% were common between semen and urine [23]. Notably, variability in semen microbiome composition can be the result of environmental factors, personal hygiene habits, and age [27]. 

With regard to semen microbiome composition, studies have shown that Proteobacteria and Actinobacteria predominantly occur in the semen of healthy men, along with Bacteroidetes and Firmicutes [28]. One study exploring the semen microbiome showed that among healthy study participants, the microbiome was dominated among others by genera of *Pelomonas*, *Propionibacterium*, *Bosea*, *Xylanimicrobium*, *Pedomicrobium*, *Phyllobacterium*, and *Mycobacterium*, and species of *Propionibacterium acnes* and *Corynebacterium simulans* [29]. Another pyrosequencing study has identified 21 genera of bacteria in semen, including *Ralstonia*, *Corynebacterium*, and *Lactobacillus* among others [21]. Researchers concluded that the observed between-person variability in bacterial species supports that the semen microbiome is unique and influenced by each person’s genetic and environmental factors [21].

**Figure 1:**
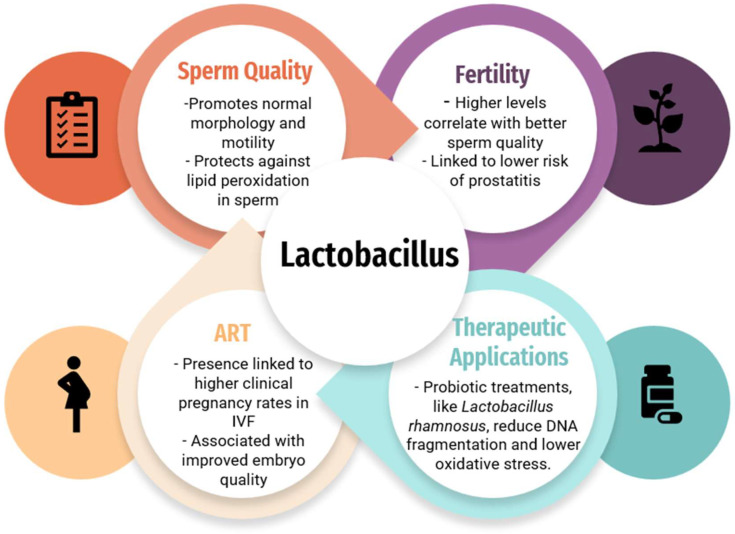
The impact of the *Lactobacillus* species on fertility, sperm quality, and ART outcomes, and therapeutic applications of the *Lactobacillus species* [25,34,35,36].

Comparisons between patients with prostatitis and healthy controls revealed a higher abundance of *Lactobacilli*, particularly *L*. *iners*, in healthy individuals [26]. In line with these findings, *Lactobacillus*-predominant semen has been reported to have higher quality [25] and lower levels of *Lactobacillus,* or elevated levels of *Neisseria* and *Klebsiella pneumoniae*, which have been linked to conditions like hyperviscosity and oligoasthenoteratozoospermia (Figure 1) [12,13]. In accordance with Monteiro et al., a study comparing the semen from vasectomy and non-vasectomy participants using NGS technology found that *Lactobacillus* is commonly associated with high-quality sperm and a lower risk of prostatitis [30]. In conjunction with such findings, it has been proposed that female tract *Lactobacilli* may help prevent sperm lipid peroxidation and, thus, also maintain sperm quality during sperm migration and fertilization (Figure 1) [31]. Inversely, an increased presence of *Lactobacillus* in semen has also been linked to potential fertility issues. Such dysbiosis can compromise the stability of beneficial microorganisms, leading to the growth of opportunistic pathogens [28]. In addition, the role of *L. gasseri* in semen is debated [30]. While some research indicates that it is predominantly found in vaginal secretions and is linked to positive outcomes in IVF treatments [32], other studies have suggested that *L. gasseri* may reduce sperm motility in vitro [33].

With regard to the effect of a vasectomy on the semen microbiome, research has indicated that overall, the abundance of *Lactobacillus* does not significantly change following a vasectomy [30]. However, *L. gasseri* may be notably higher in samples from vasectomized men compared to those from non-vasectomized men [30]. In addition, *Corynebacterium* abundance has been reported to increase after a vasectomy [30]. According to another study, the vasectomy influenced the microbiome, and *Finegoldia* was higher in uncircumcised men [15]. It remains, though, unclear whether these differences in semen microbiome between vasectomized and non-vasectomized men reflect a direct effect of a vasectomy on the semen microbiome or are the result of differential sexual habits.

### 2.2. Semen Microbiome in Cases of Male Infertility

Research on the semen microbiome has revealed a complex relationship between microbial composition and male fertility, with studies showing varying results (Table 1). Amato et al. found no significant differences in bacterial genera between infertile patients and healthy controls [37], and Alfano et al. [38] demonstrated that the amount of bacterial DNA is inversely proportional to the levels of normal spermatogenesis.

In men who are infertile due to azoospermia, a reduced relative abundance of Bacteroidetes and Proteobacteria in testis has been reported [38], while an increased relative abundance of Bacteroidetes and Firmicutes has been reported by another research group [28], collectively revealing the controversial role of Bacteroidetes in sperm production and semen with regard to male infertility. Additional research has highlighted the role of specific bacterial genera in influencing sperm quality. A relative abundance of *Mycoplasma* and *Ureaplasma* was shown to be greater among azoospermic study participants [32], while a greater abundance of *Neisseria* and *Klebsiella pneumonia* has been implicated in excessive sperm viscosity and oligoasthenoteratozoospermia [28]. In a study implementing 16S rRNA sequencing for taxonomy and shotgun metagenomics to analyze semen samples from 25 infertile men and 12 healthy controls [15], infertile men showed increased *Aerococcus* and decreased *Collinsella*. *Prevotella* was negatively correlated with sperm concentration, while *Pseudomonas* was positively correlated with motility [15]. In addition, anaerobes were more common in men with varicocele [15]. In agreement with these results, *Prevotella*, in particular, has been consistently linked to poor sperm quality across multiple studies [14,39,41]. Baud et al. [39] and Gdoura et al. [41] identified *Prevotella* as the predominant genus in the semen of infertile men, often associated with abnormal sperm samples. In addition, in asthenozoospermia and oligoasthenozoospermia cases, researchers have reported several compositional aberrations, including a relative increase in genera *Ralstonia*, *Ureaplasma*, *Bacteroides*, *Aerococcus*, *Anaerococcus*, *Stenotrophomonas*, *Delftia*, *Finegoldia*, *Corynebacterium*, and *Lactobacillus*, along with a relative decrease in genera *Pelomonas*, *Propionibacterium*, *Bosea*, *Sphingomonas*, *Phyllobacterium*, *Pedomicrobium*, *Xylanimicrobium*, and *Mycobacterium*, [29]. Lastly, *Morganella morganii* species have been implicated in increased cell apoptosis and necrosis in semen samples [44].

Hou et al. [21] found that, although there were no significant differences in overall microbial composition between fertile and infertile men, *Anaerococcus* was negatively correlated with sperm quality. Furthermore, the presence of *Corynebacterium*, which can act by reducing sperm motility, has been noted to increase in infertile men [7]. In addition, bacteria like *Lactobacillus*, *Bacteroides*, and *Delftia* were found to systematically affect sperm morphology and DNA, even leading to mitochondrial disruption [29]. The acrosome, a membranous organelle located under the membrane of the head of the spermatozoon that contains various hydrolytic enzymes responsible in aiding the sperm penetration of the oocyte coats, can also be affected by the semen microbiome, and many Gram-negative bacteria cause changes in the membranes through peroxidation [45]. *Escherichia coli*, *Proteus mirabilis*, and *Proteus vulgaris* in semen are linked to lower sperm motility, acrosome damage, DNA fragmentation, and cell death in animal studies [43,46,47], while a higher prevalence of *Pseudomonas* compared to *Lactobacillus* correlates with increased sperm viscosity and oligoasthenoteratozoospermia [12].

Conversely, specific bacterial species have been shown to correlate with favorable sperm parameters. Beneficial bacteria like *Lactobacillus*, which are more prevalent in healthy sperm, are often linked to good sperm quality. For example, *Lactobacillus* was more frequently found in samples with normal sperm morphology [39], while a decrease in the relative abundance of *Lactobacillus* in semen has been observed in cases with excessive viscosity and oligoasthenoteratozoospermia [28]. It is also suggested that bacteria like *Propionibacterium* and *Atopobium* along with *Lactobacillus*, present in normal sperm, may help maintain semen quality and protect against the negative effects of Gram-negative bacteria [14].

Overall, the role of the semen microbiome in male factor infertility remains controversial, both regarding the total bacterial load in semen and the presence of specific bacterial genera and species. Several different bacteria have been implicated, but consistency in results between individual studies is missing. Based on the available research data, *Bacteroides*, *Prevotella*, *Ureaplasma*, *Corynebacterium*, and *Lactobacillus* are more likely to play a role and warrant further investigation. Nonetheless, the involvement of other bacterial genera and species in male infertility cannot be excluded, and the underlying pathogenetic mechanisms that link the microbiome with male infertility remain unclear.

### 2.3. Semen Microbiome and Sperm DNA Fragmentation

Sperm DNA Fragmentation (SDF) encompasses a variety of DNA alterations, including point mutations, deletions, duplications, and single or double-stranded breaks. Recent meta-analyses have highlighted the critical role of SDF in male infertility [48]. Emerging evidence links changes in the semen microbiome with alterations in sperm characteristics, including SDF, driving interest in this area of metagenomic research [49].

To support the hypothesis that the semen microbiome directly affects SDF, studies have shown that regulating the semen microbiome has beneficial effects. A clinical study showed that a three-week treatment with *Lactobacillus rhamnosus* and *Bifidobacterium longum* improved semen quality in asthenozoospermic men, significantly reducing SDF and oxidative stress (Figure 1) [50]. Additionally, antibiotic treatment has been shown to increase sperm concentration and decrease SDF in men with genitourinary infections [51,52,53].

### 2.4. The Semen Microbiome and Its Interaction with the Female Reproductive System

The semen microbiome interacts with the vaginal and endometrial microbiome, a process that may explain to an extent how the semen microbiome affects conception rates and pregnancy outcomes. Studies of semen separation techniques used in intrauterine insemination have demonstrated that semen contains protective antimicrobial factors, potentially involved in lower rates of adverse pregnancy outcomes [54]. In addition, longer exposure to a partner’s sperm has been associated with increased regulatory T cells in women, which may enhance pregnancy rates by moderating maternal anti-fetal immune responses [55]. The semen microbiome may also influence the vaginal microbiome, affecting the sperm’s ability to cross the cervical barrier [56]. The impact of intercourse on the reproductive tract microbiome of infertile couples has also been studied. PCR analysis revealed that *Ureaplasma parvum*, found in a high percentage of women and their partners with inflammatory prostatitis, can cause shifts in the microbiome, potentially affecting fertilization and pregnancy success [57]. Lastly, the interaction between the semen microbiome and the endometrial microbiome can influence implantation and placental development [22].

The interplay between the semen microbiome and the female reproductive system is crucial for understanding fertility and pregnancy outcomes, and the role of the immune system has also been explored. The high diversity of the semen microbiome compared to the low diversity of the vaginal microbiota can cause a major shift in the latter [13]. This disturbance changes the *Lactobacilli*-dominated microbiota to a bacterial vaginosis (BV)-like microbiota. This BV alteration may induce changes in cytokine profiles, thus resulting in differences in immunogenicity, affecting implantation or early embryo development. The final outcome could be difficulties with fertilization and complications such as recurrent spontaneous abortions. Probiotic administration has been explored as a means to address such imbalances. Treatment with *Lactobacillus casei rhamnosus Döderleini* has shown to improve antigenicity, with increased human leukocyte antigen (HLA) class I expression in fertile couples and couples experiencing recurrent pregnancy abortions, though HLA class II increased only in fertile individuals [19]. Additionally, HLA class I and HLA class II expression in spermatozoa was found to be reduced in men whose partners experienced recurrent spontaneous abortions, suggesting a potential role for HLA in stimulating the female immune response necessary for successful pregnancy [58]. Recent advances in sequencing technologies have also revealed the epigenetic complexity of semen and its role in regulating early embryonic development [59]. Seminal plasma’s role in preparing the maternal reproductive tract for embryo implantation underscores the importance of the semen microbiome in reproductive success [60].

This evolving understanding of the semen microbiome’s role in fertility and reproductive success suggests that analyzing the semen microbiome and its metabolic environment could pave the way for personalized reproductive medicine and improved fertility management. Thus, a new therapeutic target has been identified, namely manipulating the semen microbiome. Probiotics have shown to be successful in treating female reproductive concerns and thus have the potential to optimize the male microbiome as well [61]. Data from multiple studies suggest that microbiome-based interventions—such as probiotics and antimicrobial peptides—could play a significant role in improving male fertility, especially in the cases of unexplained infertility or male factor infertility linked to microbiome imbalances. Probiotics may be able to reset reproductive tract dysbiosis and enhance sperm motility and integrity by reducing oxidative stress and inflammation, which are common contributors to infertility [62]. In a pilot study of men with asthenozoospermia treated with the same oral antioxidant probiotic strains, sperm motility was improved, and DNA fragmentation was decreased [50]. The administration of a probiotic and prebiotic combination for six months was significantly associated with improved sperm count, ejaculate volume, sperm concentration, progressive motility, and the progression of typical forms in a randomized controlled trial [63]. Probiotics have also been explored, as they have regulatory effects on the immune system by modulating pattern recognition receptors such as Toll-like receptors (TLRs), which influence inflammatory responses and antibody production [40]. Furthermore, antimicrobial peptides, such as semenogelins [42], and secretory leukocyte protease inhibitors [64] play a significant role in semen’s immune defense, which may influence microbial control and overall semen health. Thus, enhancing antimicrobial peptide production could be a promising therapeutic approach, provided these peptides are not spermicidal.

### 2.5. Impact of Semen Microbiome on Outcomes of Assisted Reproduction Techniques (ARTs)

Historically, research on the semen microbiome has primarily focused on sperm parameters, but recent studies suggest that it also impacts assisted reproduction outcomes. Specific bacterial species in semen have been linked to embryo quality after in vitro fertilization (IVF). In particular, the classes Alphaproteobacteria and Gammaproteobacteria have been associated with poorer embryo quality, while the family Enterobacteriaceae, with better embryo quality [65]. However, it is important to note that other factors like infertility etiology, stimulation protocols, and oocyte numbers may influence these results [65].

With regard to IVF outcomes, the interaction between the semen microbiome and the female reproductive system is also critical. In a study of 951 IVF couples, dysbiosis in semen and vaginal or cervical samples was linked to a lower clinical pregnancy rate (19.5%) compared to couples with only vaginal infections (36.2%) [20]. Furthermore, other research groups have found notable differences in the microbiomes of semen and vaginal samples between couples that achieved clinical pregnancy after IVF and those that did not [32]. Semen from cases with a subsequent clinical pregnancy showed higher colonization by *Lactobacillus jensenii* and *Faecalibacterium*, and lower levels of Proteobacteria, *Prevotella*, and *Bacteroides* (Figure 1) [32]. In vaginal samples, *Lactobacillus gasseri* was more prevalent in cases with subsequent clinical pregnancy, while *Bacteroides* and *Lactobacillus iners* were less common. These findings suggest that enhancing the *Lactobacillus genus* in IVF couples could improve outcomes [32]. Finally, specific genital pathogens have been associated with IVF failure. Pathogens like *Enterococcus faecalis*, *Ureaplasma urealyticum*, *Mycoplasma hominis*, *Gardnerella vaginalis*, and *Escherichia coli* were more common in couples with IVF failure [66]. Notably, the group including *E. faecalis*, *U. urealyticum*, and *M. hominis* was found significantly more in IVF failure cases (36.3%) compared to successful cases (16.7%) [66]. This highlights the importance of screening and managing microbial infections to improve ART outcomes.

In contrast to the data regarding the role of semen microbiome in ART outcomes, not all studies support its role. Amato et al. [37] found no significant difference in the semen microbiome between couples with successful and failed intrauterine insemination (IUI). Recent research, which studies the vertical transmission of microbes into embryo culture media (ECM) and its association with assisted reproductive outcomes, shows that microbes can vertically transmit from semen and follicular fluid to embryo culture media, and semen was the main source of contamination in conventional IVF cases. Strong correlations were found between specific microbial taxa in semen and sperm quality; however, no significant association was found between the microbiomes of ECM, semen, and follicular fluid and ART outcomes [67].

## 3. Discussion

This review highlights the association between the composition of the semen microbiome and infertility, as revealed by data arising from the molecular analyses of the semen microbiome. The semen microbiome has been shown to differ in infertile male patients as compared to healthy controls, to associate with sperm DNA fragmentation, and to affect IVF outcomes. Studies that manipulate the semen microbiome had promising results, which support the hypothesis of an etiologic association between the semen microbiome and reproductive health. Nonetheless, consistency in results between individual studies is low, which is the main limitation in the interpretation and generalization of these data.

In an attempt to explain the low consistency between studies, which examine the semen microbiome, several limitations and potential sources of bias attributed to the molecular techniques used have been identified. Firstly, molecular techniques offer high sensitivity and specificity but cannot differentiate between live and dead microbial cells, as they detect bacterial genetic material irrespectively of bacterial viability. Thus, data arising from molecular analyses may reflect not only the current microbial load but also the recent history of the semen microbiome in the host, or even a transient presence of microorganisms [39]. Importantly, microbial colonization within the host is a dynamic process that can change over time and, therefore, the results of semen microbiome studies may change over time. Repeated semen sampling could aid in addressing this potential source of error. Furthermore, the proper use of positive and negative controls is crucial in these studies. Positive controls help detect biases and drawbacks attributed to the next-generation sequencing platform used [68], while negative controls are essential for identifying and excluding contaminants [39]. In addition, parallel sampling for urine and the gut microbiome could enhance accuracy, but many studies lack sample collections of urine or rectal samples. 

The bioinformatics and statistical analyses performed may also contribute to the observed heterogeneity in individual studies’ results. To begin with, the probability of a type I error is expected to be high in studies that associate compositional differences in microbial communities and health outcomes, owing to the very large number of statistical comparisons performed. Even in the case of studies that have applied statistical corrections to account for multiple testing, these might not be sufficient. In this case, several false positive results may have been reported. Furthermore, the power of the reviewed studies may be insufficient, as power calculations were not consistently reported, either a priori or a posteriori, which would lead to false negative results. Lastly, methodological differences, such as the next-generation sequencing platform and statistical methods used, may contribute to the observed heterogeneity in results.

As of now, studies on the semen microbiome have predominantly relied on abnormal sperm parameters as markers of infertility. However, it is important to note that traditional sperm analysis does not fully capture the functional capabilities of spermatozoa, such as the acrosome reaction, zona pellucida binding, and successful fertilization. While the conventional methods of sperm analysis provide valuable insights, they may not be sufficient for a comprehensive assessment of male fertility, particularly in relation to the complex interactions with the semen microbiome. The examination of pregnancy outcomes might, thus, provide better insight into the role of the semen microbiome in human reproduction.

The presence of microbial genetic material in the male reproductive system, as detected through the molecular analysis of semen, can significantly disrupt metabolic processes. However, whether this disruption negatively affects normal functions and ultimately impacts reproductive health depends on a complex interplay of genetic, immunological, and environmental factors. Understanding how microbial genetic material influences physiological functions requires the careful monitoring of these changes, which are driven by the transcriptional activity of the microbes. To advance this field, future research should prioritize transcriptomics studies that investigate the relationship between the microbial transcriptome and reproductive health. The transcriptome and proteome of sperm samples include RNAs and proteins produced by microbial genetic material, contributing to the cells’ metabolic profiles. Since proteins determine cellular phenotypes, conducting comprehensive studies with a broader range of proteins and larger patient populations may be essential for effectively translating these findings into clinical practice.

## 4. Conclusions

Research on the semen microbiome, utilizing sequencing technologies, has consistently shown that semen has its own microbiome, which is far more complex than previously thought, encompassing a diverse range of bacteria with both beneficial and detrimental effects on sperm quality and reproductive outcomes. Emerging evidence suggests that specific bacterial genera, such as *Prevotella* and *Lactobacillus*, play critical roles in influencing sperm parameters and fertility, while pathological microbial colonization can lead to inflammation, sperm DNA fragmentation, and reduced reproductive success. While the traditional methods of sperm analysis provide valuable insights, they fall short in capturing the full scope of microbial influence on sperm functionality. Future research should aim to integrate metagenomics, transcriptomics, and proteomics to further elucidate the role of the semen microbiome in fertility, with a focus on translating these findings into effective clinical interventions and personalized treatment strategies for improving reproductive health.

## Figures and Tables

**Table 1:** Bacterial genera in the semen microbiome and their impact on male fertility.

Bacterial Genus/Species	Presence in Fertile/Infertile Men	Impact on Sperm Quality	Associated Conditions
BENEFICIAL EFFECT			
*Lactobacillus (e.g., L. iners, L. gasseri)*	Present in fertile men	Associated with higher sperm quality [39]	Reduced risk of prostatitis [30]; better ART outcomes [32]; *L. iners* lower in oligoasthenoteratozoospermia [28]
Proteobacteria	Present in fertile men, increased levels in certain infertile cases	Varied impact, potentially negative in high amounts	Reduced in azoospermia [38]; lower levels may be beneficial for successful IVF outcomes [40]
Bacteroidetes	Present in fertile men	Potentially beneficial, but controversial in infertility [28]	Reduced in azoospermia [38]
DETRIMENTAL EFFECT			
*Neisseria*	Increased in infertile men	Associated with hyperviscosity [28]	Linked to oligoasthenoteratozoospermia [28]
*Klebsiella pneumoniae*	Increased in infertile men	Linked to sperm apoptosis and reducing sperm motility	Associated with hyperviscosity [28]
*Prevotella*	Increased in infertile men	Associated with oligozoospermia and obesity-associated asthenozoospermia [14,39,40]	Higher levels are associated with lower sperm counts [15] and motility issues [14,39,41]
*Corynebacterium*	Very increased in infertile men	May reduce sperm motility and morphology [29]	Increased post-vasectomy [30]
*Mycoplasma and Ureaplasma*	Present in infertile men	Negative impact on sperm motility and quality [41]	Linked to azoospermia [32] and genitourinary infections [41]; common in couples with IVF failure [42]
*Escherichia coli*	Present in infertile men	Linked to acrosome damage and DNA fragmentation [43]	Associated with increased SDF [43]
*Pseudomonas*	Present in infertile men	Associated with higher motility [15] but increased viscosity [12]	Associated with oligoasthenoteratozoospermia [12]
*Ralstonia*	Increased in infertile men	Linked to reduced sperm quality [29]	Associated with asthenozoospermia [29]
*Stenotrophomonas*	Present in infertile men	Linked to reduced sperm quality [29]	Present in asthenozoospermia [29]

## Data Availability

No new data were created or analyzed in this study. Data sharing is not applicable to this article.

## Supplementary Materials

The following supporting information can be downloaded at: https://www.mdpi.com/article/10.3390/ijms26041446/s1.
