# Supplementary material for: Semen Microbiome, Male Infertility, and Reproductive Health
Source: Int J Mol Sci. 2025 Feb 9;26(4):1446. doi: 10.3390/ijms26041446 (PMC11854939; doi:10.3390/ijms26041446)
Supplement: Supplementary file 1 [file ijms-26-01446-s001.zip › ijms-3463156-supplementary.pdf]

Search Strategy and search results:

For this review, we considered as eligible all original research studies that examined the human semen microbiome, in relation to male fertility and reproductive success. With regard to study design, prospective, retrospective, and cross-sectional cohort studies were eligible along with case-control studies and analyses embedded in clinical trials. If studies utilized only culture-based techniques to analyze the semen microbiome, they were excluded from this review, as were the studies that were published in language other than English.

The literature search was originally performed in PubMed on January 2023 and was updated on January 2024. In our search algorithm, we used both keywords (“microbiome”, “microbiota”, “microflora”) and Mesh Terms ("microbiota"[MeSH]) to describe the microbiome. Furthermore, we specified the semen and the relevant outcomes using the following keywords: “sperm”, “semen”, “genital”, “male”, “reproduction”, “fertility”, “infertility”,”IVF”, “ICSI”, “pregnan*”, “oligosperm*”, “oligozoosperm*”, “azoosperm*”, “teratosperm*”, and “teratozoosperm*”. The aforementioned keywords and Mesh terms were combined with the use of appropriate Boolean operators (OR, AND). We additionally applied a filter to safely exclude animal studies from our search results (NOT (animals [mh] NOT humans [mh])). Lastly, the references of all included studies were screened for potentially relevant research papers that were not captured by our search algorithm (“snowball” procedure).

During the initial search, 693 titles and abstracts were detected. After applying the afore-presented search strategy, 68 studies met our inclusion and exclusion criteria and are discussed in this review.
